# Supplementary material for: Studies in vitro and in vivo of phage therapy medical products (PTMPs) Targeting Clinical Strains of Klebsiella pneumoniae belonging to the clone ST512
Source: Antimicrob Agents Chemother. 2025 Apr 23;69(6):e01935-24. doi: 10.1128/aac.01935-24 (PMC12135510; doi:10.1128/aac.01935-24)
Supplement: Table S1 — Value of the count of CFU/mL and PFU/mL at time 0, 12 and 24 h. [file aac.01935-24-s0001.pdf]

SUPPLEMENTARY MATERIAL

Table S1. Value of the count of CFU/mL and PFU/mL at time 0, 12 and 24 h.

	0 h	12 h	24 h
**CAC_Kpn1**	4,8E+06 CFU/mL	2.1E+05 CFU/mL	2.5E+08 CFU/mL
	1.95E+08 PFU/mL	8.2E+08 PFU/mL	6.4E+09 PFU/mL
**CAC_Kpn1_ad**	4.8E+06 CFU/mL	4.7E+03 CFU/mL	5.4E+04 CFU/mL
	5.4 E+08 PFU/mL	4E+08 PFU/mL	3.6E+08 PFU/mL
**CAC_Kpn2 (Strain B)**	2.25E+6 CFU/mL	9.4E+07 CFU/mL	2.3E+08 CFU/mL
	4.8E+5 PFU/mL	2.5E+08 PFU/mL	4.5E+08 PFU/mL
**CAC_Kpn2_ad (Strain B)**	2.25E+6 CFU/mL	9.6E+07 CFU/mL	2.25E+08 CFU/mL
	4.1E+05 PFU/mL	3.3E+08 PFU/mL	2.3E+08 PFU/mL
**CAC_Kpn2_ad (Strain B) + 1/2 Mer**	2.25E+06 CFU/mL	7.4E+03 CFU/mL	6.3E+05 CFU/mL
	4.1E+05 PFU/mL	7.3E+06 PFU/mL	6.3E+07 PFU/mL
**CAC_Kpn2 (Strain C)**	5.35E+06 CFU/mL	1.5E+08 CFU/mL	4.3E+08 CFU/mL
	2.5E+06 PFU/mL	5.7E+08 PFU/mL	3.4E+08 PFU/mL
**CAC_Kpn2_ad (Strain C)**	5.35E+06 CFU/mL	1.6E+08 CFU/mL	6.7E+08 CFU/mL
	5.53E+06 PFU/mL	3.5E+08 PFU/mL	1.3E+09 PFU/mL
**CAC_Kpn2_ad (Strain C) + 1/2 Mer**	5.4 E+06 CFU/mL	3.4E+05 CFU/mL	1.6E+06 CFU/mL
	5.5E+06 PFU/mL	7.0E+06 PFU/mL	1.5E+09 PFU/mL
